# Original observations of *Desmozoon lepeophtherii*, a microsporidian hyperparasite infecting the salmon louse *Lepeophtheirus salmonis*, and its subsequent detection by other researchers

**DOI:** 10.1186/1756-3305-4-231

**Published:** 2011-12-13

**Authors:** Mark A Freeman, Christina Sommerville

**Affiliations:** 1 Institute of Biological Sciences & Institute of Ocean and Earth Sciences, University of Malaya, Kuala Lumpur, 50603 Malaysia; 2 Institute of Aquaculture, University of Stirling, Stirling FK9 4LA UK

**Keywords:** *Desmozoon*, *Paranucleospora*, *Lepeophtheirus*, Microsporidian, Copepod, opportunistic, Immunocompromised, Grilse

## Abstract

A microsporidian hyperparasite, *Desmozoon lepeophtherii*, of the parasitic copepod *Lepeophtheirus salmonis *(salmon louse), infecting farmed Atlantic salmon (*Salmo salar*), was first discovered in the west of Scotland in 2000. Heavily infected salmon lice are easily recognised as they have large opaque inclusions distributed throughout the body. The prevalence of salmon lice with visible signs of microsporidiosis can be up to 10% of the population from certain farm sites. The microsporidian was also isolated from the host Atlantic salmon suggesting it may have a two host life cycle. The authors believe that the infection in immunocompetent salmon may be latent, becoming acute during periods of infection with another pathogen or during sexual maturation. Since its first discovery in Scotland, *Desmozoon lepeophtherii *has been subsequently reported from Norway, and more recently from the Pacific coast of North America.

## Background

The microsporidian hyperparasite *Desmozoon lepeophtherii *was originally discovered in Scotland during a PhD programme carried out at the Institute of Aquaculture, University of Stirling, between October 1998 and September 2002. The primary objective of the research was to screen parasitic copepods (salmon lice) infecting farmed and wild Atlantic salmon for potential microbial candidates that could be evaluated for use in alternative control strategies. The study was jointly funded by the Biotechnology and Biological Sciences Research Council (BBSRC) and the Scottish Salmon Growers Association (SSGA), now the Scottish Salmon Producers' Organisation (SSPO).

### Original discovery of a hyperparasitic microsporidian from Lepeophtheirus salmonis

A microsporidian was first isolated from the salmon louse, *Lepeophtheirus salmonis*, infecting farmed Atlantic salmon from Western Scotland in December 2000 [1]. The characteristic gross pathology observed in microsporidian-infected salmon lice had, however, been observed during previous lice collections from farmed salmon in 1999 and early 2000, but was not confirmed as a microsporidian hyperparasite until December 2000 [1]. *Lepeophtheirus salmonis *exhibiting the clinical signs of microsporidiosis, i.e. numerous opaque internal inclusions distributed throughout the body, were found at three of fifteen farm sites sampled between 13/10/1998 and 15/05/2002 [1]. The number of *L. salmonis *infected with the microsporidian per farm site varied between 1% and 10%, with an average of 5% of adult female *L. salmonis *having the microsporidian infection when present at the farm site. Infection with the microsporidian in female salmon lice was often associated with poorly developed or aborted egg strings [1,2]. When the microsporidian was present in adult female *L. salmonis*, adult male lice from the same population were also infected with a similar prevalence [1]. One site that was known to harbour microsporidian-infected *L. salmonis *was sampled 17 times over a 22 month period, but no clear seasonal trend in occurrence emerged [1]. However, the maximum percentage prevalence of 10% was considered to be a conservative estimate as it only included *L. salmonis *that were showing visible signs of infection, and many more lice may have had less advanced infections.

As heavily infected *L. salmonis *showed advanced signs of pathology, such as the inability to produce viable egg strings, a patent application was made in 2001. The patent "Microbiological control of sea lice" United Kingdom patent GB2371053, international patent PCT/GB02/00134 included the microsporidian among other candidates for use in alternative control strategies for sea lice.

The microsporidian was initially characterised using both molecular and ultrastructural studies which were documented in the PhD thesis, completed in 2002 [1]. Subsequently, the molecular identification of the microsporidian was published in a peer reviewed journal article in 2003 [2] and the partial ribosomal DNA sequence data (1411 bp including the ITS region) was deposited in GenBank (Feb 2002) with the accession number AJ431366. The ultrastructure, including a full taxonomic description of the new genus and species, *Desmozoon lepeophtherii*, followed in another peer reviewed article in 2009 [3] with specimens collected during January 2001, consisting of two EM grids showing spores and developmental stages deposited as hapanotypes in the Natural History Museum, London.

Once the molecular identification had been completed, and the microsporidian from *L. salmonis *was shown to be related to the salmonid microsporidian parasite *Nucleospora salmonis*, farmed Atlantic salmon from the same farm location were tested using a specific PCR for the presence of the microsporidian parasite. The microsporidian DNA was successfully amplified from four fish kidney tissues and a contiguous sequence of 860 bp, including the ITS region, was constructed. This sequence matched the original salmon lice sequence, commencing from base 260 to base 1120, and shared 99.4% similarity, differing in only 5 bases and was considered to be from the same microsporidian parasite [1]. Additional Atlantic salmon from the same farm site (those with two or more visibly infected salmon lice) were further sampled for the microsporidian, which was successfully detected from the kidney, liver, heart, gill and peripheral blood mononucleocytes [1]. DNA sequencing of these samples showed 100% identity with the 860 bp of data from the first four fish kidneys.

*Desmozoon lepeophtherii*, from the type location in Scotland, was resampled from *L. salmonis *and re-sequenced in early 2010 to resolve the minor differences seen between the original sequence from *L. salmonis *and the subsequent one obtained from Atlantic salmon tissues. The new sequence, compiled from four infected *L. salmonis*, extended the original 1411 bp of data to include the complete small subunit (16S) to 1787 bp. During this sequence update in March 2010, it became clear that the initial sequence obtained from *L. salmonis *contained some very minor sequencing errors, and the correct sequence was the one reported from Atlantic salmon in the PhD study [1]. These minor sequencing errors (9 bases of 1411 (0.64%)) in the initial sequence were attributed to the relative lack of experience of the PhD student (MAF), at the time, in using slab gel DNA sequencers.

### Discovery of Desmozoon lepeophtherii in Norway

Shortly after the formal description of *Desmozoon lepeophtherii *from *L. salmonis *in Scotland had been published in 2009 [3], a new microsporidian genus and species description, *Paranucleospora theridion*, infecting both *L. salmonis *and Atlantic salmon from Norway, was published in 2010 [4]. In this publication the authors mentioned the microsporidian DNA sequence obtained from Scottish *L. salmonis *in 2003, but surprisingly failed to include it in their phylogenetic analyses. However, they concluded that the microsporidian from Scottish *L. salmonis *was "now identified with *P. theridion*" and claimed it to be a synonym. The present authors assumed that this had been a timing oversight, by the Norwegian group, due to the fairly recent publication of the *D. lepeophtherii *description [3]. Yet, a second manuscript on the same microsporidian from Norway, by the same group, was published in 2011 [5], which also failed to recognise the validity of the *D. lepeophtherii *publication. In that manuscript, they refer to an earlier publication naming *P. theridion *[6], which predates the *D. lepeophtherii *publication [3], and claimed that *P. theridion *was the first published name for this parasite, to endorse their continued use of the name in the 2011 manuscript [5]. The publication they refer to [6] is an article in a non peer-reviewed 'popular scientific journal' (Pers. Comm. Christine Elgen; Naturen), hence not appropriate for the naming of a novel scientific species. Furthermore, the article in question did not actually attempt to describe the microsporidian or even mention it as a new species, but did refer to it as *P. theridion*, and cite the forthcoming descriptive manuscript [4] as 'submitted'.

### Discovery of Desmozoon lepeophtherii in the Pacific

In a recent study of sea lice from the Pacific coast of North America, three different microsporidian parasites were sampled and identified using partial ribosomal DNA sequences and ultrastructural studies [7]. One, exclusively isolated from *L. salmonis *on cultured Atlantic salmon, was identified as *Desmozoon lepeophtherii *and is the first report of *D. lepeophtherii *from the Pacific Ocean. The other two microsporidians were not restricted to *L. salmonis *and were found in other parasitic copepods sampled from different fish. They clustered within a clade of microsporidians that infect other crustacean hosts and were not closely related to *D. lepeophtherii *and the Enterocytozoonidae. *Desmozoon lepeophtherii *occurred only in *L. salmonis *from cultured Atlantic salmon and was not found in other *Lepeophtheirus *spp. and no evidence of *D. lepeophtherii *was observed in the salmon host [7]

### Pathology of Desmozoon lepeophtherii in Atlantic salmon

During the original discovery of *D. lepeophtherii *(between 1999 and 2002) from *L. salmonis *on farmed Atlantic salmon in Scotland, and its subsequent isolation from fish tissues, no new epizootics were apparent and no unexplained increases in fish mortalities were reported. Fish that were confirmed as positive for the presence of the microsporidian using diagnostic PCRs appeared externally normal except for an occasional mild gill pallor. However, the kidneys of some fish, which were confirmed as strongly positive with the diagnostic PCR, were moderately enlarged and discoloured to a greenish-grey and contained numerous white flecks. Histopathology of the kidney tissue showed moderate hyperplasia in the renal interstitium, with numerous mitotically active immature white blood cells. Heart tissue showed endocardial activation and hyperplasia with the occasional hypertrophy of myocardial nuclei. Other tissues appeared normal except for the occasional reticular cell proliferation of ellipsoids in the spleen [1]. It was also reported that the microsporidian was far more readily detected (strongly positive in diagnostic PCRs) in fish showing secondary sexual characteristics (grilse), suggesting that grilse fish were more heavily infected with the microsporidian [1].

Nylund et al. [4] state that *D. lepeophtherii *has been associated with up to 80% mortality in commercial farms producing Atlantic salmon in Norway, but, unfortunately, do not include any supporting data. In addition, they suggest that *D. lepeophtherii *is associated with the disease conditions proliferative gill inflammation (PGI), pancreas disease (PD), heart and skeletal muscle inflammation (HSMI), and cardiac myopathy syndrome (CMS) in Atlantic salmon [4]. But, the following year, Nylund *et al*. [5] report that *D. lepeophtherii *only appears as a possible primary agent in cases with high mortality in connection with PGI, and not the other conditions, which all occurred in the absence of *D. lepeophtherii *[5].

In higher vertebrates, microsporidian and other fungal infections as primary pathogens are largely asymptomatic and typically result in few clinical signs; exposure to microsporidia in humans is common and chronic microsporidiosis is not linked to any clinical manifestation in healthy individuals [8]. However, microsporidia are known to be opportunistic parasites that can proliferate considerably at times when the host is immunosuppressed due to existing conditions such as HIV/AIDS and others [9]. As such they can be considered to be latent parasites that opportunistically use periods of host immunosuppression to proliferate, causing the diseased condition microsporidiosis [8,10]. We believe that a similar situation exists with *D. lepeophtherii *infection in Atlantic salmon. *Desmozoon lepeophtherii *may be a common microsporidian parasite of farmed Atlantic salmon with only chronic or even latent infection levels in the fish host, resulting in no obvious clinical signs. However, like other microsporidian parasites, *D. lepeophtherii *is highly opportunistic in nature and can proliferate to cause acute disease, with associated clinical signs, when the host is immunocompromised. Therefore, during the onset of other diseases, such as heart and skeletal muscle inflammation (HSMI), pancreas disease (PD), proliferative gill inflammation (PGI) and cardiac myopathy syndrome (CMS), it is likely that a latent microsporidian parasite will multiply and cause additional clinical signs or exacerbate the ones associated with the primary pathogen. Furthermore, during the original description of *D. lepeophtherii*, it was reported that infection rates seemed to be far higher in grilse salmon [1]. Grilse salmon are known to be far more susceptible to certain parasitic infections such as *Kudoa thyrsites*, where infection rates in grilse fish may reach 70% but are below 10% in non-sexually maturing fish [11]. Therefore, it is possible that the immunosuppression associated with sexual maturation in salmonid fish may predispose them to infection and allow opportunistic latent parasites to become problematic and cause complicated multifactorial etiologies for some disease conditions.

## Conclusion

*Desmozoon lepeophtherii *is the valid name for the microsporidian parasite (Family: Enterocytozoonidae) found infecting both *L. salmonis *and Atlantic salmon in Northern Europe and the North Pacific. This formal binominal nomenclature was assigned in a peer-reviewed publication and published in 2009 [3], before the Norwegian description of *P. theridion *in 2010 [4]. The earlier publication, in the non peer-reviewed popular journal, Naturen [6], cannot be considered to be a valid description, as suggested by Nylund *et al*. 2011 [5]. *Desmozoon lepeophtherii *is therefore the senior synonym and is registered on the World Register of Marine Species (WoRMS) as such, with *Paranucleospora theridion *noted as a junior synonymised taxon. http://www.marinespecies.org/aphia.php?p=taxdetails&id = 565086

Nylund *et al*. [4] described the parasite as having two cycles of development in the Atlantic salmon and one in the salmon louse. Transmission mechanisms between Atlantic salmon and *L. salmonis *have not been identified but both hosts are thought to be involved in the life cycle [4]. It is very interesting that *D. lepeophtherii *has been reported from the Pacific and that it has only been found infecting *L. salmonis *from farmed Atlantic salmon. *Lepeophtheirus salmonis *from the Pacific are believed to be genetically different from *L. salmonis *in the Atlantic and they are suggested to have independently co-evolved with Pacific and Atlantic salmonids respectively, showing a level of genetic divergence consistent with an independent evolution over the last 2.5-11 million years [12]. Therefore, it would be extremely interesting to know whether Atlantic salmon, that were introduced to the Pacific for aquaculture purposes, arrived with asymptomatic *D. lepeophtherii *infections and were able to infect native (Pacific) *L. salmonis *populations that presumably had not been infected by *D. lepeophtherii *for millions of years, or if the infected salmon lice in the Pacific are 'Atlantic' *L. salmonis*.

## Competing interests

The authors declare that they have no competing interests.

## Authors' contributions

The PhD project referred to in this study [1] was undertaken by MAF under the supervision of CS. This communication was discussed at length by MAF and CS and the manuscript drafted by MAF. All authors read and approved the final version of the manuscript.
